# Estimating microhaplotype allele frequencies from low-coverage or pooled sequencing data

**DOI:** 10.1186/s12859-023-05554-z

**Published:** 2023-11-03

**Authors:** Thomas A. Delomas, Stuart C. Willis

**Affiliations:** 1 Agricultural Research Service, United States Department of Agriculture, National Cold Water Marine Aquaculture Center, 483 CBLS, 120 Flagg Road, Kingston, RI 02881 USA; 2 Hagerman Genetics Laboratory, Columbia River Inter-Tribal Fish Commission, Hagerman, ID USA

**Keywords:** Low-depth whole genome sequencing, Skim-seq, Pool-seq, Microhaplotype, Genotype panel design

## Abstract

**Background:**

Microhaplotypes have the potential to be more cost-effective than SNPs for applications that require genetic panels of highly variable loci. However, development of microhaplotype panels is hindered by a lack of methods for estimating microhaplotype allele frequency from low-coverage whole genome sequencing or pooled sequencing (pool-seq) data.

**Results:**

We developed new methods for estimating microhaplotype allele frequency from low-coverage whole genome sequence and pool-seq data. We validated these methods using datasets from three non-model organisms. These methods allowed estimation of allele frequency and expected heterozygosity at depths routinely achieved from pooled sequencing.

**Conclusions:**

These new methods will allow microhaplotype panels to be designed using low-coverage WGS and pool-seq data to discover and evaluate candidate loci. The python script implementing the two methods and documentation are available at https://www.github.com/delomast/mhFromLowDepSeq.

**Supplementary Information:**

The online version contains supplementary material available at 10.1186/s12859-023-05554-z.

## Background

As the cost of obtaining genetic information has decreased, more applications for this information have been created. Genotypes are now used across medicine, forensics, agriculture, and natural resource management to inform decisions [1–5]. For a subset of applications, such as genomic selection, pedigree/relationship inference, and genetic stock identification, it is often necessary for a given program to genotype a large number of individuals [6–8]. To make these applications cost-effective, only a moderate number of loci (often a few hundred) can be genotyped [9–11], which limits the statistical power of the panel.

Genotyping microhaplotypes instead of SNPs can increase the variability of a given genetic panel without increasing the number of loci genotyped. A microhaplotype locus contains multiple SNPs that are close enough to be genotyped in the same sequencing read, and so genotyping a microhaplotype locus via sequencing (e.g., amplicon sequencing) uses the same resources as genotyping a locus with one SNP using the same technology. Because microhaplotypes can display more than two alleles, they can achieve higher variability than a biallelic SNP. The use of microhaplotype panels has been demonstrated to increase power compared to SNP panels for applications in forensics [12], genetic stock identification [13], and pedigree inference [14, 15]. While their utility for genotype imputation has not been directly evaluated, imputation is known to be more accurate when informed by larger numbers and/or more variable SNPs [9, 10, 16, 17]. Microhaplotypes can therefore be expected to outperform single SNPs in this important use case as well.

Several strategies have been used to identify and estimate allele frequencies for microhaplotypes in order to design genotyping panels. One opportunistic strategy is to select single SNPs for an amplicon sequencing panel and incorporate any additional SNPs that happen to be in the targeted amplicons [18]. A more targeted method is to use the reduced-representation technique restriction site-associated DNA sequencing (RAD-seq) to identify candidate loci as this allows samples to be genotyped for a common set of loci and SNPs to be phased over short distances using read-based phasing [, 14, 19, 20]. The main drawback to using RAD-seq is that it only covers a small portion of the genome [21]. For projects that require information on a larger fraction of the genome or simply require a larger set of candidate loci to select from, reduced-representation techniques are not applicable. In these cases, whole genome sequencing (WGS) and phasing can be used to identify microhaplotypes [22, 23] but has not been widely applied to non-model organisms. Presumably, this is because of the prohibitive cost to sequence a large enough number of individuals to estimate allele frequencies in every population of interest.

When information on a large fraction of the genome is needed but individual information is not, two main techniques have been previously utilized. The first is to sequence a mixed DNA sample derived from a single population, often referred to as pool-seq [24]. Alternatively, low-coverage WGS data from many individuals can be analyzed with methods that account for genotype uncertainty [25]. Both techniques allow genome-wide information to be collected at a lower cost than high-depth WGS. Computational techniques have been developed to infer population SNP allele frequencies from these data types [26–30]. For example, Kim et al. [26] describe fitting a mixture model and obtaining maximum-likelihood estimates of allele frequency by integrating over individual genotype uncertainty. Raineri et al. [27] consider pool-seq data and describe a Bayesian model that allows the use of priors reflective of different experimental situations (e.g., when the ancestral allele is known). Additionally, when the set of all possible haplotypes is known, methods exist to infer haplotype frequencies from these data types [31–33]. However, there are no existing tools to estimate haplotype, including microhaplotype, allele frequencies without additional information. To address this, we here describe and validate methods to estimate microhaplotype allele frequencies from both pool-seq and low-coverage WGS data. These methods will enable the cost-effective design of microhaplotype panels for applications that benefit from highly variable loci.

## Methods

We developed two related methods for estimating microhaplotype allele frequencies. The “individual” method addressed low-coverage WGS datasets where reads can be assigned unambiguously to individuals. The second, “pool” method addressed pool-seq datasets where reads are not able to be assigned to individuals. Both methods utilized mixture models to infer allele frequencies. In the individual method, the individual was the unit of observation, and the genotype was a latent variable. Component weights were genotype frequencies and were linked to allele frequencies by assuming Hardy–Weinberg equilibrium (HWE). In the pool method, the read was the unit of observation and the allele that it was derived from was a latent variable. Component weights were allele frequencies.

### Individual method

When sequencing reads can be assigned to individuals but the depth of sequencing is too low to definitively call genotypes, methods have previously been used that take genotype uncertainty into account to infer SNP allele frequencies through a maximum likelihood approach [26]. We extend this approach to the case of microhaplotypes.

Let $\uppi$ be a vector of genotype frequencies for one locus and *R*_*i*_ be the set of all sequencing reads for one individual (individual *i*) that cover one or more SNPs in the target locus. The likelihood of $\pi$ given *R*_*i*_ (probability of *R*_*i*_ given $\pi$) was described by the mixture model equation

1 $${\text{P}}\left( {R_{i} |\uppi } \right) = \mathop \sum \limits_{j}^{K}\uppi _{j} P\left( {R_{i} |z = G_{j} } \right),$$

where *K* was the number of genotypes, *z* was the unknown genotype of the individual, and ${G}_{j}$ was genotype *j*. Assuming independence, the likelihood across all individuals was calculated as the product of all individual likelihoods. The probability of a set of reads given a genotype, $\mathrm{P}\left({R}_{i}|z={G}_{j}\right)$, was calculated as described by Eqs. (1) and (2) in Edge et al. [34]. This likelihood uses the probability that a sequencing call is incorrect, which we calculated according to standard probability arguments from a user supplied probability that the base was incorrect prior to sequencing (e.g., PCR error in library prep, which was set at 0.01 for the current study) and the Phred score that represents the probability the base was called incorrectly during sequencing. This model was fitted using an expectation–maximization algorithm and allele frequencies were linked to genotype frequencies by assuming HWE.

### Pool method

Given sequencing reads that could not be assigned to individuals, we assumed that reads were drawn from the entire population of alleles at random. We utilized a mixture model to represent the sequencing reads, with the specific allele that a given read originates from as a latent variable [31]. The mixture proportions represented the population allele frequencies and the likelihood of a read given an allele was calculated as described by Eq. (1) in Edge et al. [34]. This likelihood uses the probability that a sequencing call is incorrect which was calculated as described above. The model for an individual read can be represented by Eq. 1 with terms redefined: *R*_*i*_ was a single read, $\uppi$ was a vector of population allele frequencies, *K* was the total number of alleles, *z* was the unknown allele that the read originated from, and ${G}_{j}$ was allele *j*. Assuming independence between reads, the likelihood given all reads was calculated as the product of the individual likelihoods. This model was fit using an expectation–maximization algorithm and there was no assumption of HWE.

### Pruning of alleles considered

In both methods described above, a key parameter was the number of different alleles at a given locus. If all possible alleles at a microhaplotype were considered, this would grow exponentially with the number of SNPs. When analyzing low-coverage WGS data, the number of possible genotypes was also important, and this would grow faster than the number of alleles. The number of potential alleles/genotypes would quickly become computationally prohibitive if all possibilities were considered. However, strong linkage between SNPs in a given microhaplotype causes many of the possible alleles to be non-existent. To manage the computational burden, we needed a simple, efficient method of removing alleles with frequency of zero from consideration.

The method implemented here was to iteratively estimate allele frequencies within successively larger subsets of a given locus while removing alleles with estimated frequency close to zero at the end of each iteration. For a locus containing *y* total SNPs, only the first *x* SNPs were considered in the first iteration. The model was fit and alleles with estimated frequency below *c* were removed from consideration. In the second iteration, the first 2*x* SNPs were considered with haplotypes for the first *x* SNPs being restricted to only those retained at the end of the first iteration. Additional iterations were performed (adding up to *x* SNPs with each iteration) until all *y* SNPs were considered in the final iteration. In the final iteration, no alleles were dropped regardless of estimated frequency as this would not increase computational efficiency. An example can be found in Additional file 1. For the current study, the values of *x* and *c* were set to 1 and 0.001, respectively.

### Implementation

These methods were implemented as a python script that takes as input one or more bam files, a file containing the known positions of substitution SNPs, and optional user specified parameters. Methods of SNP discovery for both data types considered here exist [25, 27, 35–37], and so we focused only on estimation of allele frequency given a known set of SNPs. The implementation only considers substitution SNPs (not indels or complex variants). Both methods described here were applied using a window of 60 bps to define microhaplotype loci. The window advanced until at least one new SNP was included to prevent considering a locus that is a subset of another locus. To avoid situations that are computationally prohibitive, estimates were not made for any loci where one or more SNP had a depth of zero or where the number of alleles being considered was greater than 128 or 256 for the individual or pool methods, respectively. The python script utilized the packages Pysam (https://www.github.com/pysam-developers/pysam) [38, 39], numpy [40], and numba [41] and is available at https://www.github.com/delomast/mhFromLowDepSeq.

### Method evaluation

#### Overview

These methods were tested on three publicly available paired-end sequencing datasets from non-model organisms. Two were WGS datasets containing 25 Pacific oysters *Crassostrea gigas* from Weihai, China [42] and 53 Atlantic salmon *Salmo salar* from the St. John River strain [43] while the third was a RAD-seq data set containing 96 Pacific lamprey (80 from the Willamette River in Oregon, USA and 16 from the Yakima River in Washington, USA) [44]. Results for the three datasets were highly similar, and so figures presented here are for the oyster WGS dataset while corresponding figures for the other datasets are available as supplementary material.

For each dataset, we first called genotypes and performed short-range, read-based phasing. The resulting genotypes were used to calculate allele frequencies and expected heterozygosity for microhaplotypes with a length of 60 bp using a custom java program (https://www.github.com/delomast/mhFromLowDepSeq/tree/main/testVCFcalc) relying on the htsjdk API (https://www.github.com/samtools/htsjdk). These statistics calculated from the full data are referred to throughout as “observed” allele frequencies and expected heterozygosity. Imitation low-coverage sequencing datasets with mean individual depth of 0.1, 0.5, 1, and 2 were then created by randomly subsampling aligned reads using samtools [39]. All individuals in a given dataset were downsampled using the same frequency (probability) of retaining a read to preserve relative differences in sequencing depth between individuals. To simulate pool-seq data, individual identifiers (read groups) on reads in these downsampled datasets were ignored. The two estimators described here were run with the four low-coverage datasets to estimate allele frequencies and expected heterozygosity. Expected heterozygosity was calculated by subtracting the sum of squared allele frequencies from one. We then compared the estimates to the observed values with loci binned by the number of reads (across all individuals) that informed the estimate. This binned number of reads is not strictly sequencing depth as some reads may only cover a subset of the SNPs in a given locus. Estimates from all four levels of downsampling were combined into each bin. In cases where a locus appeared more than once in a bin, one estimate was randomly selected. Only loci with 2 or more SNPs and observed expected heterozygosity greater than 0.1 were considered during the evaluation as the focus of these methods is on variable microhaplotypes. When assessing estimates of allele frequency, one allele that had an observed frequency greater than 0.01 was selected at random from each locus in each bin. Bias and mean square error were assessed by calculating the error as “observed – estimated” frequency for that allele.

#### Pacific oyster WGS data

Whole genome sequencing data from 25 Pacific oyster *Crassostrea gigas* samples from Weihai, China described by Li et al. [42] was downloaded from the NCBI SRA. The reads were adapter and quality trimmed using TrimGalore (https://www.github.com/FelixKrueger/TrimGalore) which is a wrapper for cutadapt [45] then aligned with bowtie2 [46] in end-to-end mode with –score-min L,0,-0.4 and all other parameters taking default values to the reference genome cgigas_uk_roslin_v1 (RefSeq GCF_902806645.1) [47]. Alignments were filtered to only include reads with both mates aligning concordantly to chromosome NC_047565.1 using samtools [39]. Reads were restricted to one randomly selected chromosome as this provided sufficient data to evaluate the methods. Duplicates were removed using Picard tools (http://broadinstitute.github.io/picard/) and samples were genotyped using GATK HaplotypeCaller followed by GenomicsDBImport and GenotypeGVCFs with default settings [48, 49]. The resulting genotypes were filtered to only include biallelic SNPs with depth of 5 or greater, genotype quality of 10 or greater, variant quality of 100 or greater, minor allele frequency greater than 0.05, and a missing genotype rate of 25% or less. Genotypes were phased using WhatsHap [50, 51] with default settings. The observed allele frequencies and expected heterozygosity were calculated using only genotypes whose phase (within the locus) was able to be unambiguously determined. The aligned reads were subsampled, and the two novel estimators were applied as described above.

#### Atlantic salmon WGS data

Whole genome sequencing data from 53 Atlantic salmon *Salmo salar* samples from the St. John River strain described by Gao et al. [43] were downloaded from the NCBI SRA. This data was processed as described above for the oyster data set except that reads were aligned to the reference genome USDA_NASsal_1.1 (GenBank: GCA_021399835.1) [52] and then filtered to only include chromosome CM037941.1.

#### Pacific lamprey RAD-seq data

Due to the relative scarcity of whole-genome sequencing datasets containing a large number of samples in non-model species, we additionally tested the methods using a RAD-seq data set for Pacific lamprey *Entosphenus tridentatus* described by Hess et al. [44]. From this dataset, a subset of 96 individuals (80 from the Willamette River in Oregon, USA and 16 from the Yakima River in Washington, USA) were used. To identify and genotype SNPs, this dataset was analyzed with the dDocent pipeline [53] and the ETRf_v1 (GenBank: GCA_014621495.2) reference genome [54]. The genotypes were filtered to only include those with variant quality of 100 or greater, minor allele frequency greater than 0.05, and a missing genotype rate of 25% or less. Genotypes were then phased with WhatsHap [50, 51] using default parameters.

A major way that single-enzyme RAD-seq data deviates from whole genome sequencing data is that in paired-end RAD-seq data, one read from each pair starts at a restriction enzyme cutsite. This results in multiple reads that all start at the same position. In a WGS dataset, one would expect reads to be closer to randomly distributed. To account for this, we excluded any SNPs from consideration that were within one read length of a cutsite in the reference genome using a custom python script (https://www.github.com/delomast/mhFromLowDepSeq/blob/main/filterLamp.py). This left only SNPs that would be sequenced by the read in a pair that did not start at the cutsite. Because the insert size was variable in this library, the positions of these reads are variable and therefore a closer approximation of what is expected in a WGS dataset.

## Results

Discovery of SNPs in the oyster, salmon, and lamprey datasets yielded 809,951, 139,518, and 28,257 SNPs, and grouping these into microhaplotypes gave 356,732, 25,455, and 7,387 loci containing two or more SNPs within a 60 bp window, respectively. After filtering for loci with expected heterozygosity greater than 0.1, there were 355,285, 25,245, and 7,366 loci remaining. The filtered loci had high genotyping success and the vast majority of genotypes were able to be phased (Table 1). As a result, exclusion of genotypes from calculations of the observed allele frequencies was predominantly due to missing genotypes and not due to unknown phase.

**Table 1:** Summary of loci genotyped and phasing success with WhatsHap in each test dataset

Dataset	Oyster	Salmon	Lamprey
Mean sequencing depth	18 ± 2	15 ± 3	22 ± 3
Number of SNPs	809,951	139,518	28,257
Number of microhaplotype loci	355,285	25,245	7366
Mean allelic richness	4.7 ± 2.0	3.0 ± 1.2	3.9 ± 4.1
Mean number of phased genotypes	22.10 ± 2.73	47.21 ± 4.06	82.13 ± 7.06
Mean number of unphased genotypes	0.01 ± 0.10	0.03 ± 0.25	0.05 ± 0.77

The number of microhaplotype loci, allelic richness, number of phased genotypes, and number of unphased genotypes only includes microhaplotype loci with expected heterozygosity of 0.1 or greater. Means are given as mean ± SD. Mean sequencing depth was calculated using the number of mapped reads after duplicate removal for the oyster and salmon WGS datasets and using the mean depth of called SNP genotypes for the lamprey RAD-seq dataset

**Fig. 1:**
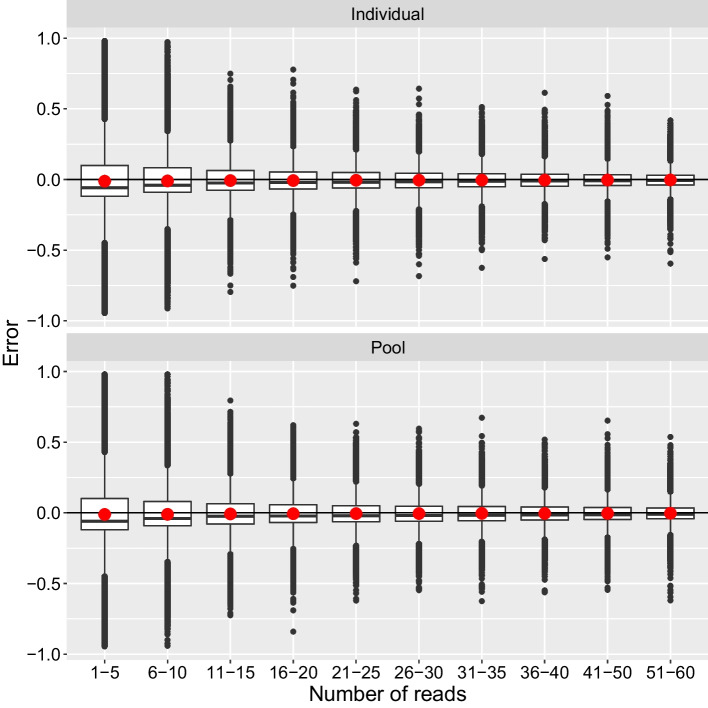
Distribution of error in estimated allele frequencies from the oyster dataset binned by the number of reads contributing to the estimate. Sample sizes in bins from left to right: 194,204, 125,410, 54,818, 81,027, 96,920, 90,745, 75,136, 63,388, 113,454, 97,540

**Fig. 2:**
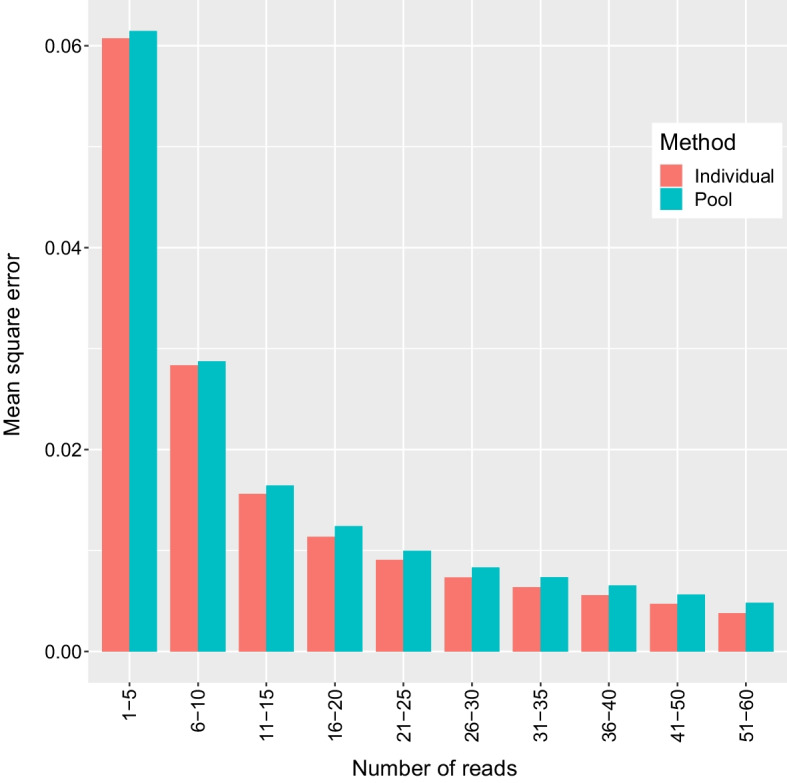
Mean square error in estimated allele frequencies from the oyster dataset binned by the number of reads contributing to the estimate. Sample sizes in bins from left to right: 194,204, 125,410, 54,818, 81,027, 96,920, 90,745, 75,136, 63,388, 113,454, 97,540

Estimates of allele frequencies were close to unbiased with mean error approximating zero (Fig. 1, Additional file 2). While the mean error was close to zero regardless of the number of reads, the median deviated from zero when very little data was available (1–5 reads). The distribution of error was less diffuse and mean square error decreased (Fig. 2, Additional file 3) when estimates were informed by more reads, but this effect was less pronounced after a minimum of 20 reads was reached. Generally, mean square error was lower with the individual method than with the pool method, but this difference was close to negligible (Fig. 2, Additional file 3).

**Fig. 3:**
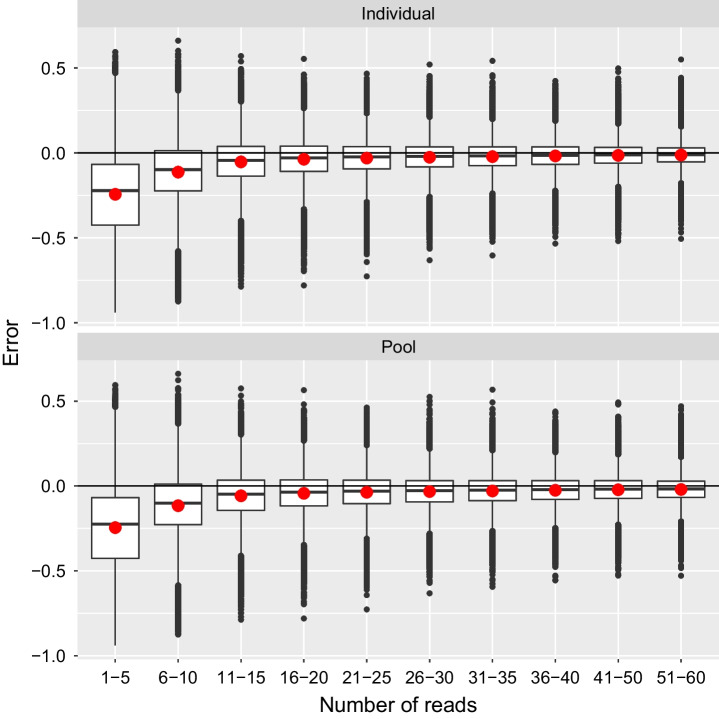
Distribution of error in expected heterozygosity from the oyster dataset binned by the number of reads contributing to the estimate. Sample sizes in bins from left to right: 194,204, 125,410, 54,818, 81,027, 96,920, 90,745, 75,136, 63,388, 113,454, 97,540

**Fig. 4:**
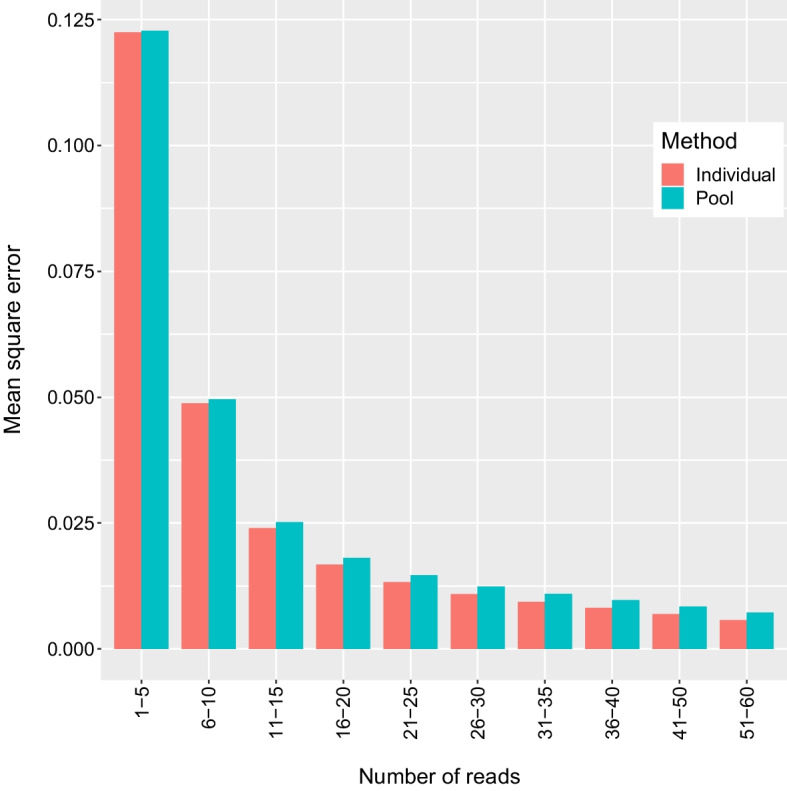
Mean square error in expected heterozygosity from the oyster dataset binned by the number of reads contributing to the estimate. Sample sizes in bins from left to right: 194,204, 125,410, 54,818, 81,027, 96,920, 90,745, 75,136, 63,388, 113,454, 97,540

Microhaplotypes are typically used when highly variable loci are desired. As such, expected heterozygosity is a useful metric when selecting microhaplotypes for a panel. We therefore examined the error in expected heterozygosity calculated from the estimated allele frequencies produced by the two methods described here. Expected heterozygosity was biased downwards at very low read numbers but this bias was minimal once 20 or more reads were used (Fig. 3, Additional file 4) corresponding to mean individual sample coverage of 0.8x, 0.4x, and 0.2 × in the oyster, salmon, and lamprey datasets, respectively. Similar to the case for allele frequencies, mean square error in expected heterozygosity decreased with increasing data and was generally lower with the individual method than with the pool method (Fig. 4, Additional file 5). However, the difference between the individual and pool methods was minor.

## Discussion

We present a new technique for discovering microhaplotype loci from low-coverage whole genome sequencing data and demonstrate that this method produces relatively unbiased and narrow estimates of allele frequencies and expected heterozygosity at sequencing depths beyond approximately 20 reads. These new methods will enable cost-effective microhaplotype panel design for organisms and populations lacking extensive individual WGS data. Many of the potential applications for microhaplotypes, including pedigree inference [14, 15], imputation for genomic selection [8], and genetic stock identification [13], involve genotyping a large number of individuals. To achieve this efficiently, smaller panels (hundreds of loci) are often designed to be population specific and genotyped through amplicon sequencing [13, 55, 56]. By allowing estimation of microhaplotype allele frequencies and expected heterozygosity from low-coverage WGS or pool-seq data, the methods developed here will allow microhaplotype panels to be designed for use in these applications, thereby increasing the statistical power achieved.

The two methods developed here were essentially unbiased for estimating allele frequencies in the tested data sets and were able to reach an approximate plateau in the decrease of mean square error with only a moderate amount of data (20–30 reads/locus, corresponding to mean individual sample coverage of 0.8–1.2x, 0.4–0.6x, and 0.2–0.3x in the oyster, salmon, and lamprey datasets, respectively). Outliers were observed with errors close to the maximum error possible at very low sequencing depths (less than 10 reads) (Fig. 1). These errors, which are presumably a result of sampling variation, can be avoided by ignoring estimates with low depth. This demonstrates that the developed methods will allow loci to be evaluated as candidates for a genotyping panel given sequencing depths recommended for pool-seq [24].

The estimates of expected heterozygosity were biased low, particularly when little data was available. This is in line with expectations based on results for estimating expected heterozygosity from individual genotypes, which is known to be biased low with bias inversely related to sample size [57]. While a correction exists for calculations based on individual genotypes [57], the derivation of a correction is not as straightforward in the current case. Fortunately, this bias was observed to be minimal with a moderate amount of data (20–30 reads/locus), and so impactfully biased estimates can be avoided by ignoring those with very low depth. It is therefore not expected to present a practical obstacle to the utilization of this technique. We also wish to be clear that with less than 20 reads per locus the method here presented was not reliable.

The individual method slightly outperformed the pool method in mean square error, but this difference was small enough that it is likely inconsequential to downstream applications. However, the datasets used in the current study were created by subsampling individual data where libraries were prepared following the same procedure. In cases where sequencing depth is more variable between individuals, the use of the individual method may correct for variance in representation. This could result in a larger difference in mean square error if HWE, which is assumed by the individual method but not the pool method, is a valid assumption.

Applications involving species relevant to aquaculture and natural resource management are particularly suited to benefit from the techniques developed here. Both fields involve a large number of species of interest, meaning the development of tools (such as genotyping panels) often does not benefit from the economies of scale present for humans, model species, and terrestrial livestock. Potential benefits from the application of modern genetic techniques to these fields have been well documented [58–63], including 22–24% increase in mean breeding value prediction accuracy from the application of genomic selection to aquaculture species [58]. The barriers cited to achieving these gains include the costs of developing genetic panels and genotyping sufficient numbers of samples [58–63], which are directly addressed by the methods presented here.

Pool-seq and low-coverage WGS have both been popular methods for cost-effective acquisition of genome-wide data, resulting in a multitude of existing datasets [24, 36, 64–68]. The methods developed here will allow these data to be reanalyzed for the purpose of designing microhaplotype genetic panels. In many cases, this will further reduce the cost of panel development by repurposing existing data.

## Conclusion

These new methods facilitate cost-effective microhaplotype panel design by allowing the use of low-coverage WGS and pool-seq data for estimation of allele frequencies and expected heterozygosity in candidate loci. The python script and documentation implementing the two methods described here are available at https://www.github.com/delomast/mhFromLowDepSeq.

### Supplementary Information

**Additional file 1**: Example of the pruning algorithm.

**Additional file 2**: Distribution of error in estimated allele frequencies.

**Additional file 3**: Mean square error in estimated allele frequencies.

**Additional file 4**: Distribution of error in estimated expected heterozygosity.

**Additional file 5**: Mean square error in estimated expected heterozygosity.

## Data Availability

The datasets analyzed in this manuscript were deposited in the NCBI SRA at the time of their original publication and are available from https://www.ncbi.nlm.nih.gov/bioproject/PRJNA394055, https://www.ncbi.nlm.nih.gov/bioproject/PRJNA559280, and https://www.ncbi.nlm.nih.gov/bioproject/PRJNA177416.

## Abbreviations

SNP: Single nucleotide polymorphism
WGS: Whole genome sequencing
HWE: Hardy–Weinberg equilibrium
