# Supplementary material for: Estimating microhaplotype allele frequencies from low-coverage or pooled sequencing data
Source: BMC Bioinformatics. 2023 Nov 3;24:415. doi: 10.1186/s12859-023-05554-z (PMC10623847; doi:10.1186/s12859-023-05554-z)
Supplement: Supplementary file 1 — Additional file 1: Example of the pruning algorithm. [file 12859_2023_5554_MOESM1_ESM.pdf]

Supplemental File 1: Example of the pruning algorithm

Consider a locus with three SNPs ($y = 3$), all three with reference allele A and alternate allele B. Let the values of x and c be 1 and 0.001, respectively. In the first iteration, we only consider the first SNP and estimate the following:

Allele	Estimated frequency
A	0.7
B	0.3

Since both estimated frequencies are greater than 0.001, we move on to the next iteration. We consider the first two SNPs and estimate the following:

Allele	Estimated frequency
AA	0.4
AB	0.3
BA	0.299999
BB	0.000001

The estimated frequency of allele BB is less than 0.001, so we remove it from consideration. We then consider all three SNPs and estimate:

Allele	Estimate frequency
AAA	0.3
AAB	0.1
ABA	0.299999
ABB	0.000001
BAA	0.299999
BAB	0.000001

These are the final estimates. Note that in the third iteration, no allele that starts with "BB" was considered. This is because it was removed from consideration after the second iteration.
