# Supplementary material for: Estimating microhaplotype allele frequencies from low-coverage or pooled sequencing data
Source: BMC Bioinformatics. 2023 Nov 3;24:415. doi: 10.1186/s12859-023-05554-z (PMC10623847; doi:10.1186/s12859-023-05554-z)
Supplement: Supplementary file 4 — Additional file 4: Distribution of error in estimated expected heterozygosity. [file 12859_2023_5554_MOESM4_ESM.pdf]

Supplemental File 4: Distribution of error in estimated expected heterozygosity binned by the number of reads contributing to the estimate. Sample sizes in bins from left to right for the oyster dataset: 194,204, 125,410, 54,818, 81,027, 96,920, 90,745, 75,136, 63,388, 113,454, 97,540, Sample sizes in bins from left to right for the salmon dataset: 15,385, 8,057, 2,747, 6,682, 8,107, 5,540, 3,893, 4,572, 10,468, 5,779. Sample sizes in bins from left to right for the lamprey dataset: 586, 2,902, 2,526, 887, 273, 213, 470, 708, 1,820, 1,853.

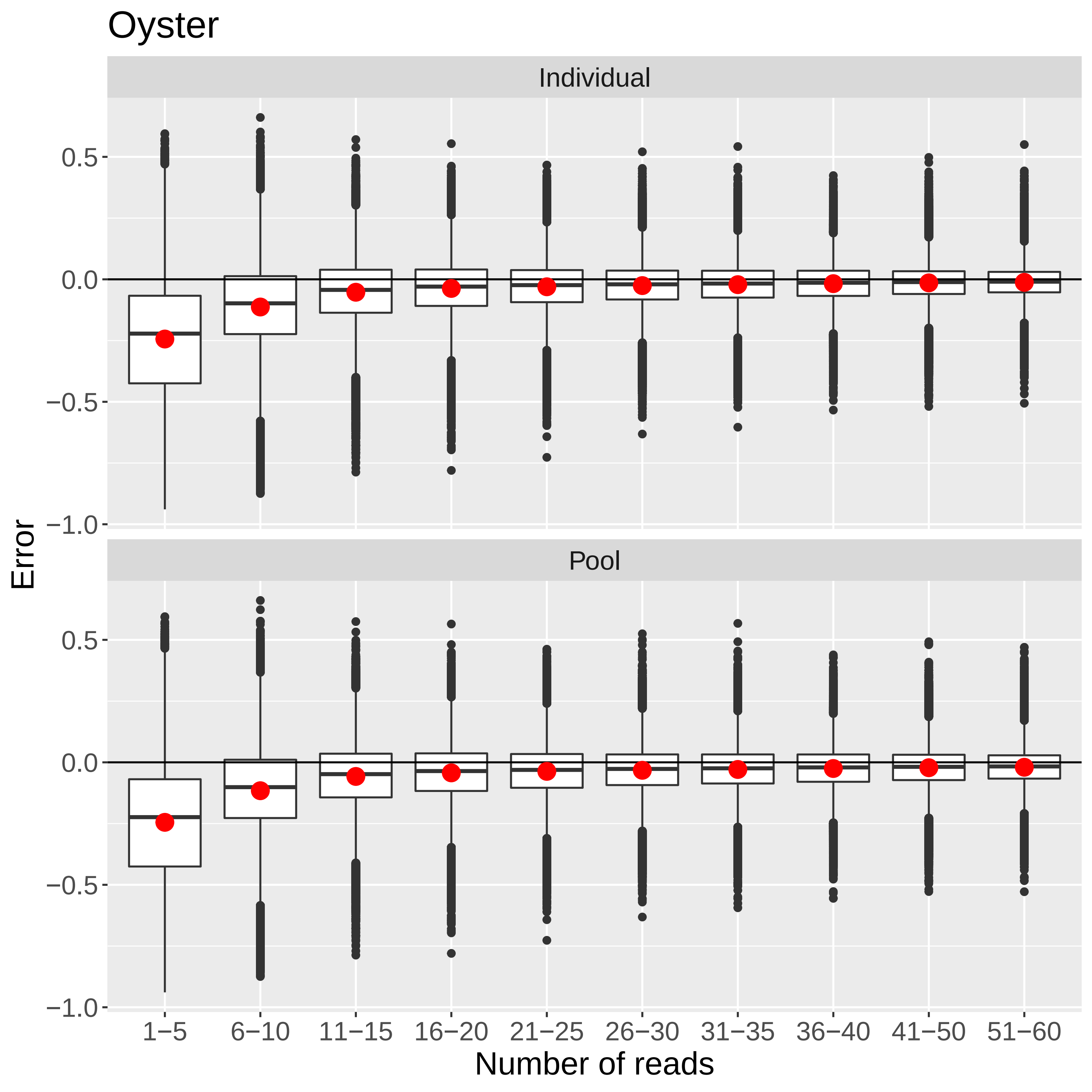
Oyster
Individual
Pool
Error
0.5
0.0
−0.5
−1.0
1−5
6−10
11−15
16−20
21−25
26−30
31−35
36−40
41−50
51−60
Number of reads

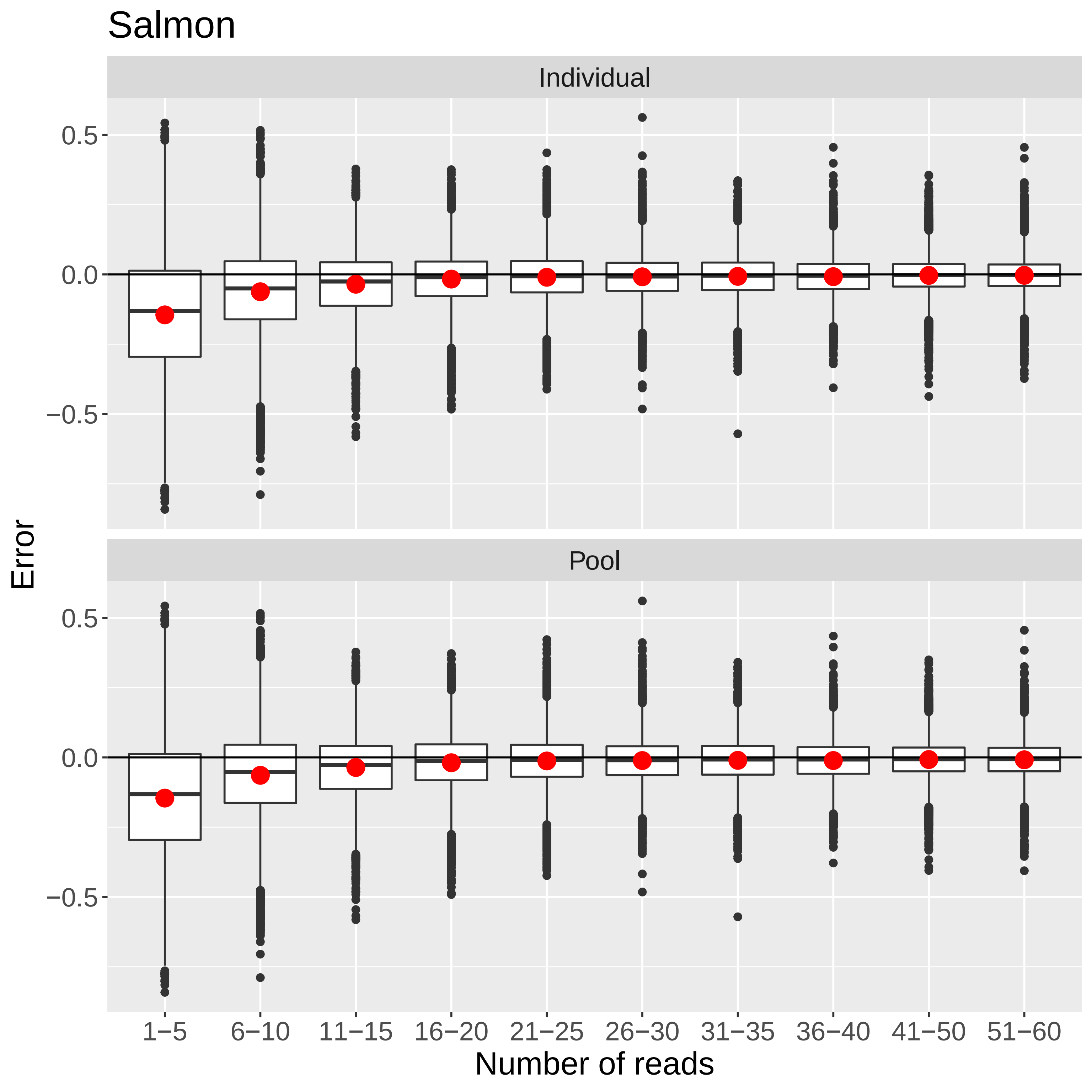

Salmon
Individual
Pool
Error
0.5
0.0
−0.5
1−5
6−10
11−15
16−20
21−25
26−30
31−35
36−40
41−50
51−60
Number of reads

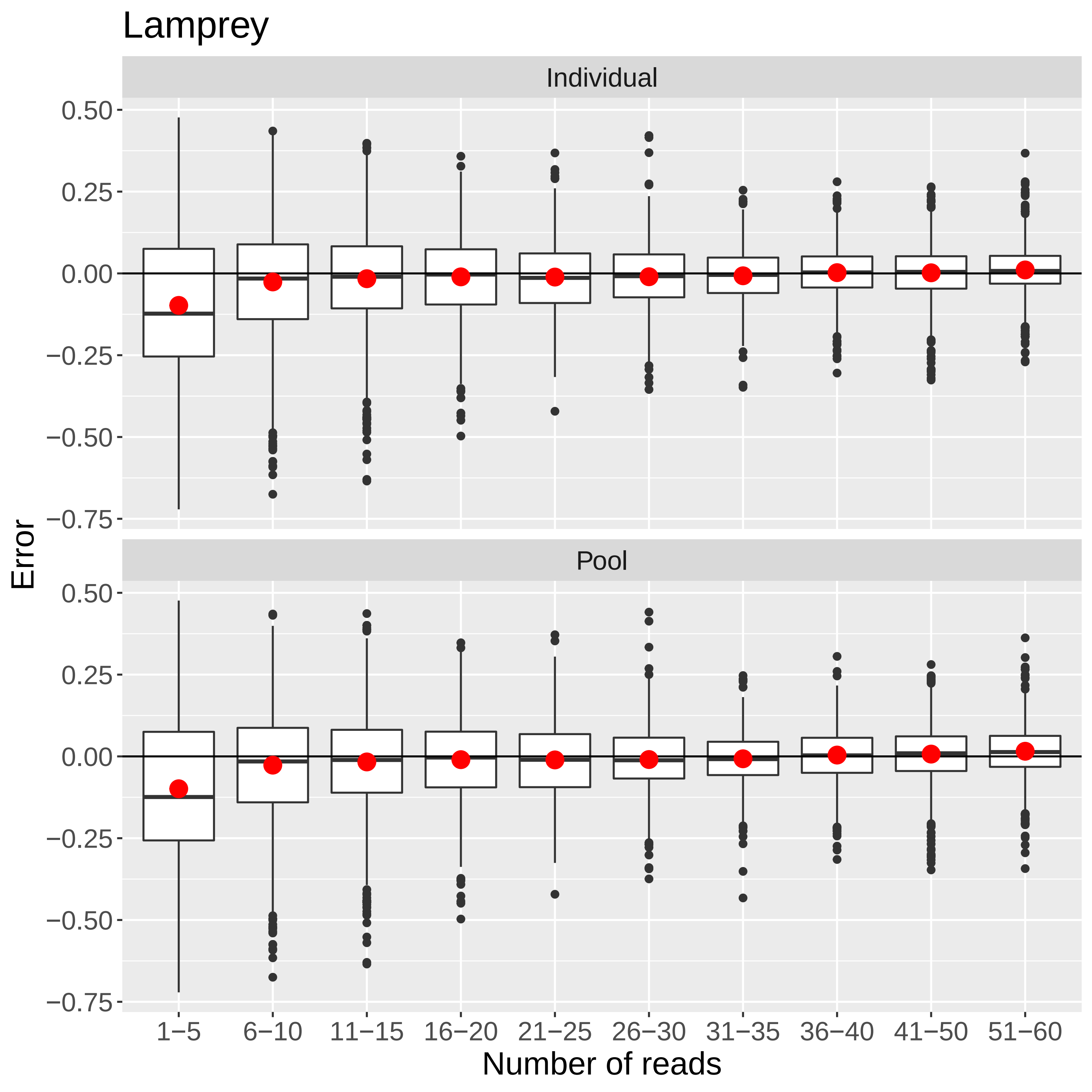

Lamprey
Individual
Pool
Error
0.50
0.25
0.00
−0.25
−0.50
−0.75
1−5
6−10
11−15
16−20
21−25
26−30
31−35
36−40
41−50
51−60
Number of reads
